# Vaccinia Virus E3 Protein Prevents the Antiviral Action of ISG15

**DOI:** 10.1371/journal.ppat.1000096

**Published:** 2008-07-04

**Authors:** Susana Guerra, Ana Cáceres, Klaus-Peter Knobeloch, Ivan Horak, Mariano Esteban

**Affiliations:** 1 Department of Molecular and Cellular Biology, Centro Nacional de Biotecnología CSIC, Campus Universidad Autónoma, Madrid, Spain; 2 Department of Preventive Medicine and Public Health, Universidad Autónoma, Madrid, Spain; 3 Abteilung Molekulare Genetik, Leibniz Institut fur Molekulare Pharmakologie, Berlin, Germany; Saint Louis University, United States of America

## Abstract

The ubiquitin-like modifier ISG15 is one of the most predominant proteins induced by type I interferons (IFN). In this study, murine embryo fibroblast (MEFs) and mice lacking the gene were used to demonstrate a novel role of ISG15 as a host defense molecule against vaccinia virus (VACV) infection. In MEFs, the growth of replication competent Western Reserve (WR) VACV strain was affected by the absence of ISG15, but in addition, virus lacking E3 protein (VVΔE3L) that is unable to grow in ISG15+/+ cells replicated in ISG15-deficient cells. Inhibiting ISG15 with siRNA or promoting its expression in ISG15−/− cells with a lentivirus vector showed that VACV replication was controlled by ISG15. Immunoprecipitation analysis revealed that E3 binds ISG15 through its C-terminal domain. The VACV antiviral action of ISG15 and its interaction with E3 are events independent of PKR (double-stranded RNA-dependent protein kinase). In mice lacking ISG15, infection with VVΔE3L caused significant disease and mortality, an effect not observed in VVΔE3L-infected ISG15+/+ mice. Pathogenesis in ISG15-deficient mice infected with VVΔE3L or with an E3L deletion mutant virus lacking the C-terminal domain triggered an enhanced inflammatory response in the lungs compared with ISG15+/+-infected mice. These findings showed an anti-VACV function of ISG15, with the virus E3 protein suppressing the action of the ISG15 antiviral factor.

## Author Summary

Modification of proteins by ubiquitin (UB) and ubiquitin-like proteins (UBL) represents a key regulatory process of innate and adaptive immune responses. Interferon-stimulated gene product 15 (ISG15) is a member of UBL molecules that can reversibly be conjugated to proteins mediating considerable antiviral response. In turn, many viruses, including poxviruses, have evolved strategies to block the antiviral and inflammatory effects of innate immune responses to keep cells alive until virus replication is completed. Here, a novel viral immune evasion mechanism that inhibits ISG15-dependent antiviral pathway is described. Vaccinia virus (VACV) pathogenesis in ISG15+/+ versus ISG15−/− mice is linked to the virus E3 protein, blocking the activity of ISG15 through its C-terminal domain. This effect was independent of PKR activation. ISG15 controls the inflammatory response regulating cytokine levels. Our findings support a new strategy for poxviruses to evade the host antiviral response through interaction of the virus E3 protein with ISG15.

## Introduction

Type I interferons (IFN-α and -β) serve a critical role in antiviral innate immunity and in modulating the adaptive immune response to infection and tumor development [1]. In response to infection or Toll-like receptor agonists, IFN is produced and consequently leads to the up-regulation of hundreds of IFN-stimulated genes (ISG) [2],[3]. One of the most highly induced genes is ISG15 that encodes a small UBL protein of 17 kDa that forms covalent conjugates with cellular proteins [4]. ISG15 is composed of two domains, each of which carries high sequence and structural similarity to UB (33 and 32% for the N- and C-terminal domains, respectively) [5],[6].

ISG15 conjugation (ISGylation) to substrate proteins occurs in a manner similar to UB conjugation by utilizing activating, conjugating and ligating enzymes to facilitate the addition of ISG15 to specific lysine residues [7]. The ISG15 activating enzyme is ubiquitin E1 like protein (UBE1L), and the E2 enzyme for UB conjugation, UbcH8, also recognizes ISG15 [8],[9],[10]. ISG15 is removed from conjugated proteins by an ISG15-specific protease, UBP43 (USP18 (UB-specific protease 18)) [11],[12],[13]. UB as a central cellular regulator and UB-mediated proteolysis also plays a regulatory role in the immune system [14],[15]. While the degradation by the proteosome generally depends on poly-UB conjugation, protein modification by ISG15 does not typically cause substrate degradation [16]. Instead it may alter the subcellular localization, structure, stability or activity of targeted proteins [17]. A large number of cellular proteins that are associated with cellular cytoskeleton, stress response and chromatin remodelling were identified as ISG15 targets. ISG15 also targets proteins that play a role in the innate antiviral response, including: PKR, MXA, STAT1, JAK1 and RIG-I [18]. ISGylation of these antiviral molecules may regulate their activity during viral infection.

ISG15 expression is almost undetectable under normal conditions but is strongly up-regulated during viral infections such as human cytomegalovirus (HCMV), herpes simplex virus (HSV), Sindbis virus (SV) and hepatitis C virus (HCV) [19],[20],[21],[22],[23],[24]. It has been speculated that the ISG15 up-regulation following viral infection is involved in different strategies of the antiviral response [25],[26]. Some viruses have developed specific strategies to counteract the activity of the IFN-stimulated genes (ISGs). The influenza B virus protein NS1B binds ISG15 and blocks protein ISGylation [27]. Furthermore, constitutive expression of ISG15 in type I IFN receptor knockout (KO) mice confers potent antiviral activity against SV. This evidence suggests that ISGylation is important for protecting cells from viral infection [21].

Previously, using cDNA microarrays we described up-regulation of ISG15 after infection of HeLa cells with the attenuated VACV strains MVA and NYVAC, an effect not observed after infection with the virulent strain WR [28],[29],[30]. Furthermore, the attenuated mutant VVΔE3L that lacks the viral early protein E3 also produces an increase in the ISG15 mRNA levels [31]. VVΔE3L is a virus that only replicates in IFN-incompetent systems exerting IFN antagonist activity [32], is nonpathogenic in the mouse model, and provides protection against a wild-type virus challenge [33],[34]. The E3 protein represses the host cell antiviral response by multiple mechanisms, including inhibition of both PKR and RNase L, two enzymes induced by IFN and whose activation triggers a global inhibition of protein synthesis and virus replication [35],[36],[37] through the phosphorylation of eIF-2α (for PKR) and breakdown of RNA (for RNase L). Significantly, once activated both PKR and RNase L produced upregulation of ISG15 messenger levels [38],[39]. E3 also blocks induction of IFN-α/β through inhibition of phosphorylation of the transcription factors IRF3 and IRF7 [40],[41] and prevention of NF-κB activation [42].

The biological significance of ISG15 mRNA induction in cultured cells after infection with the VACV mutants and its repression by the virulent WR is not known. Here we have investigated the role of ISG15 as an anti-VACV immunity factor using *in vitro* and *in vivo* systems based on MEFs and mice lacking ISG15. While in MEFs the yields of WR were slightly different between ISG15+/+ and ISG15−/− infected cells, the non-replicating VVΔE3L in ISG15+/+ cells grew more than one log better in ISG15−/− cells. Biochemical analyses showed that the E3 protein interacts with ISG15 through its carboxyl terminal domain. Repression of ISG15 with siRNAs or expression of ISG15 by a lentivirus vector in ISG15 null cells indicate that VACV replication can be controlled by ISG15 and that E3/ISG15 protein interaction is independent of the presence of PKR. In mice lacking ISG15, VVΔE3L induced stronger pathogenesis than in ISG15+/+, an effect similarly triggered by a C-terminal deletion mutant (VVE3LΔ26C). Our findings reveal a novel VACV strategy to counteract the IFN antiviral response through interaction of the virus E3 protein with ISG15.

## Results

### Expression of ISG15 is upregulated during infection of human HeLa and murine embryonic fibroblast cells with attenuated VACV strains

We and others have previously described upregulation of ISG15 transcript from gene expression profiles of HeLa cells infected with the attenuated VACV strains, VVΔE3L [31], MVA [28] and NYVAC [30]. This up-regulation was not observed in HeLa cells infected with the virulent WR [29]. Here, we have validated the transcriptional changes in ISG15 mRNA levels after VACV infection by real-time RT-PCR. As shown in Table S1, ISG15 mRNA levels at different times postinfection (p.i.) were enhanced in HeLa cells infected with the mutant viruses compared to the virulent WR, in agreement with the microarray data (not shown).

**Figure 1:**
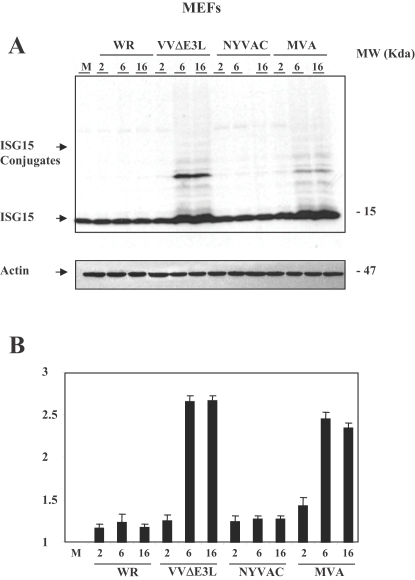
ISG15 protein levels are upregulated during infection of MEFs with the attenuated mutants of vaccinia virus. A. ISG15 protein levels after WR or VVΔE3L or MVA or NYVAC infection. MEFs were mock infected (M) or infected at 5 PFU/cell with WR or MVA or NYVAC or VVΔE3L and at the indicated times p.i, cell extracts were analyzed by Western blotting. Equal amounts of proteins were fractionated by SDS-PAGE, transferred to nitrocellulose paper, and reacted with an antibody that recognizes murine ISG15 protein. On the right, the molecular weight of the proteins in kilodaltons is indicated. Actin levels showed that the same amount of protein was loaded on the gel. Uninfected cells (M) served as control. B. Densitometric quantification of ISG15 protein in arbitrary units is indicated. The graphic represents these measurements in three independent experiments.

To correlate changes in ISG15 protein levels, we analyzed by immunoblot the levels of ISG15 in WR- or MVA- or NYVAC- or -VVΔE3L or uninfected MEFs. In agreement with the results of real time RT-PCR obtained in Hela cells, a clear increase in ISG15 protein levels was also observed in VVΔE3L- or MVA-infected MEFs cells at 6 and 16 hpi (Fig. 1A). The increase was less apparent after NYVAC infection probably because overall protein synthesis is more severely inhibited by NYVAC than MVA [30]. Moreover, the increase of ISG15 protein levels after VVΔE3L or MVA infection required de novo protein synthesis as its accumulation was prevented by cycloheximide treatment discarding the possibility that infection might increase ISG15 protein levels by enhancing protein stability (not shown). It should be noted that there is an increase in the conjugation of ISG15 to its target proteins after VVΔE3L, but reduced in levels after MVA infection (Fig. 1A). The findings of Fig. 1 establish a clear up-regulation of ISG15 by the attenuated VACV mutants.

### ISG15 has an anti-VACV role and modulates VVΔE3L replication in cultured cells

**Figure 2:**
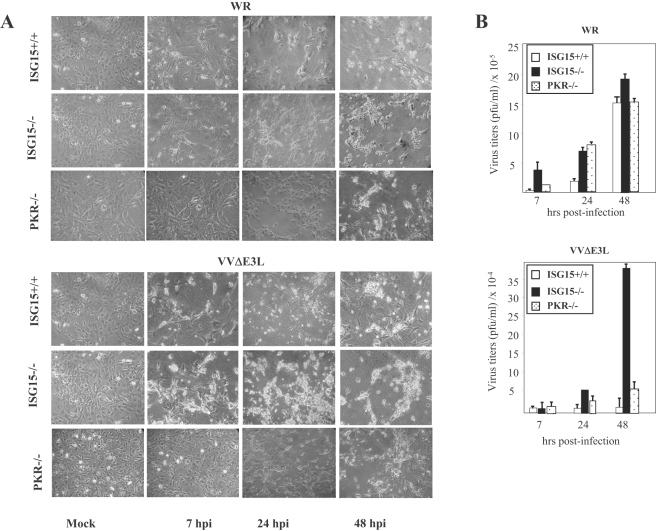
Effect of ISG15 on cytotoxicity and virus growth after infection of MEFs with virulent and E3L deletion VACV mutant viruses. A. ISG15−/−, PKR−/− or wild type cells were mock-infected or infected at 0.1 PFU/cell with WR or VVΔE3L. At different times p.i, the CPE in the cells was examined by phase-contrast microscopy. B. Virus growth of WR and VVΔE3L infected (0.1 PFU/cell) ISG15+/+, or ISG15 −/−, or PKR−/− cells. At different times cells were harvested and virus yields were determined by plaque assay for WR or by immunostaining for VVΔE3L.

Since the increase in ISG15 in Hela cells correlated with the attenuated phenotype of several VACV strains, we next examined the role of ISG15 in VACV replication using primary MEFs derived from ISG15+/+ and ISG15−/− mice. While the cytopathic effect (CPE) observed in ISG15−/− after WR infection (0.1 PFU/cell, 24 h) was similar to ISG15+/+ cells (Fig. 2A, upper panels), the CPE in ISG15−/− cells after VVΔE3L infection was markedly increased with respect to that observed in ISG15+/+ cells (Fig. 2A, lower panels). The virus titers for WR were slightly increased in ISG15−/− compared to ISG15+/+ cells (Fig. 2B), while the yields of VVΔE3L were increased in the ISG15−/− compared to ISG15+/+ cells (about 25-fold higher). The increase in virus titers correlated with increase in cellular mortality, as shown in Fig. 2A. The findings of Fig. 2 suggest that E3 expression might be suppressing ISG15 function. To define the breath of the E3 anti-ISG15 activity we analyzed the role of another antiviral factor, PKR, using MEFs derived from PKR−/− mice. Both the difference in CPE and virus yields between VVΔE3L infected ISG15−/− and PKR−/− cells were clearly distinct (Fig. 1A), indicating that the *in vitro* replication of VVΔE3L in ISG15−/− is a process independent of PKR. With PKR+/+ cells the CPE and virus yields were similar as for ISG15+/+ cells (not shown).

**Figure 3:**
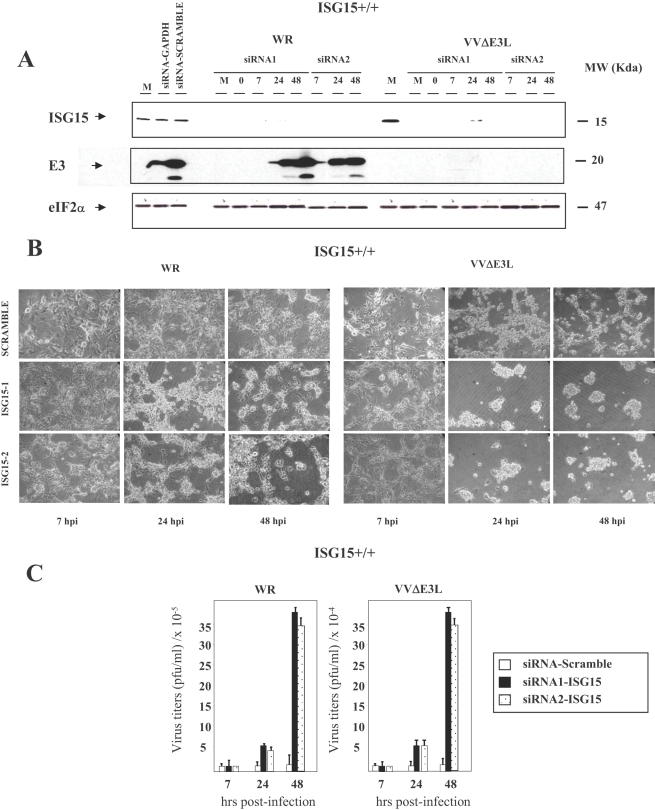
Anti VACV role of ISG15 protein expression using siRNA. MEFs were transfected with ISG15 synthetic siRNA (siRNA1 or siRNA2), scrambled siRNA (used as a negative control) and GAPDH siRNA (used as a positive control) and 24 hours after transfection, siRNA-treated and non-treated control cells were mock- or infected with different WR or VVΔE3L viruses at 0.1 PFU/cell. A. Levels of ISG15 were measured by Western-blot using a specific murine anti-ISG15 antibody. Viral protein E3 levels were measured as a control of infection and levels of eIF2α were used as a loading control. B. CPE was visualized by phase-contrast microscopy at the indicated times p.i. C. Virus growth of WR and VVΔE3L were determined by plaque assay or by immunostaining at the indicated times p.i.

To provide further evidence for a VACV antiviral role of ISG15, we used siRNA to specifically block ISG15 mRNA production. Using siPORT Amine as a transfection reagent, MEFs were transfected with two specific ISG15 siRNAs (siRNA1 or siRNA2), or with a specific GAPDH siRNA (positive control) or with a scrambled siRNA (negative control). Twenty four hours after transfection cells were infected with WR or VVΔE3L (0.1 PFU/cell), and ISG15 expression, CPE and virus titers were evaluated during the course of infection. As shown in Fig. 3A, the two ISG15 siRNAs decreased the expression of ISG15 by over 80% after 24 h of transfection (Fig. 3A). The decrease in ISG15 protein levels was accompanied by an enhanced CPE in VVΔE3L infected ISG15+/+ cells (Fig. 3B); the difference in CPE was less clear in WR-infected cells. In addition, viral titers were enhanced in silenced ISG15+/+ cells infected with WR or VVΔE3L (Fig. 3C). We also performed ISG15 mRNA inhibition with ISG15 siRNAs in PKR−/− cells and found no changes in CPE and virus yields for WR and VVΔE3L infected cells in comparison to the results obtained in PKR+/+ cells (not shown), indicating that the function of E3 protein is independent of PKR activity.

Transfection of GAPDH siRNA or a scrambled siRNA followed by infection with WR or VVΔE3L had no significant effect in either ISG15 protein level, CPE or virus production, indicating the specificity of ISG15 function. We checked that GAPDH protein levels were decreased only in the siRNA-GAPDH transfected cells, as measured by Western blot analysis (not shown). The results of Fig. 3 revealed that suppression of ISG15 protein levels leads to enhanced replication of WR and of VVΔE3L, further supporting an anti VACV role of ISG15.

### Absence of ISG15 expression is required for efficient VVΔE3L infection in murine cultured cells

**Figure 4:**
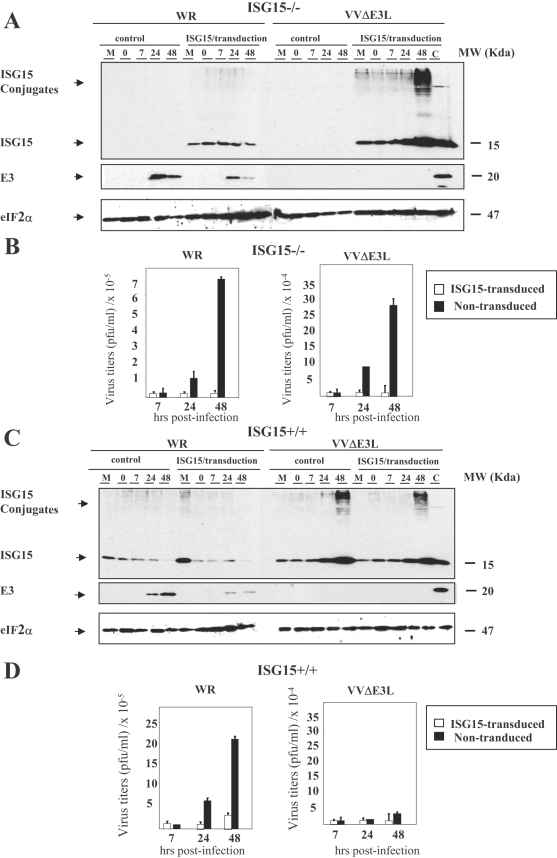
Effect of ISG15 overexpresion on virus growth after infection of MEFs with virulent and E3L deletion VACV mutant viruses. A–B. ISG15−/− MEFs were transduced with high-titer viral supernatants corresponding to the pISG15-ires-GFP retroviral vector. Levels of ISG15 were measured by Western blot using a specific murine anti-ISG15 antibody. Viral protein E3 levels were measured as control of infection and levels of eIF2α were used as a loading control. Virus growth of WR and VVΔE3L were determined by plaque assay or by immunostaining. C–D. ISG15+/+ MEFs were transduced as above. Levels of ISG15 were measured by Western blot using a specific murine anti-ISG15 antibody. Viral protein E3 levels were measured as control of infection and levels of eIF2α were used as a loading control. Virus growth of WR and VVΔE3L were determined by plaque assay or by immunostaining. Control indicates the non-transduced MEFs.

To further extend the role of ISG15 expression in VACV replication, we tested whether ectopic ISG15 expression in ISG15−/− cells leads to inhibition of WR or VVΔE3L viral growth. Using pRVISG15-ires-GFP, an optimized retroviral vector that expresses efficiently ISG15 in transduced cells, we evaluated the CPE (see Fig. S1) and viral growth of WR or VVΔE3L. Viral titrations showed that retroviral transduction of the *ISG15* gene in ISG15−/− cells result in inefficient VVΔE3L viral production, whereas non-transduced ISG15−/− cells infected with VVΔE3L were able to produce infectious viral particles (Fig. 4C, left panel). Furthermore, yields of VACV infectious virus decreased in the ISG15-transduced cells in comparison to those that do not express ISG15 (Fig. 4C, right panel). These findings demonstrate that the absence of ISG15 is essential for the productive infection of VVΔE3L and the increase in VACV production in murine cultured cells.

To characterize the effect of ISG15 over-expression in WR or VVΔE3L replication in cells with endogenous ISG15, a similar approach was carried out as before but with retroviral transduction of ISG15+/+ cells, followed by measurements of WR or VVΔE3L viral growth. As shown in Fig. 4, panels C–D, both viruses showed a decrease in infectious virus production in correlation with higher ISG15 expression levels. This experiment supports that ISG15 is a negative regulator of VACV replication.

### E3 interacts with ISG15 through its carboxy-terminal domain

**Figure 5:**
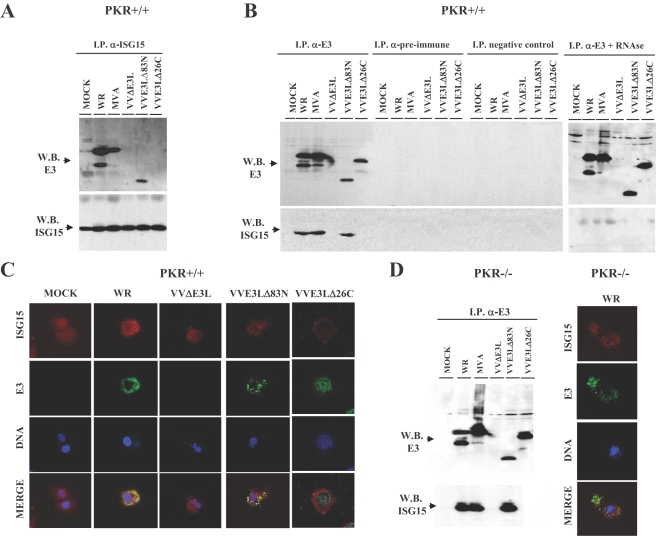
E3 interacts with ISG15 protein. A. PKR+/+ MEFs were treated with mouse IFN-α (100 units/ml) during 10 hrs and then infected with 3 PFU/cell of WR, or MVA or VVΔE3L or VVE3LΔ83N or VVE3LΔ26C for 16 hours. Cell extracts were collected at 16 hpi and immunoprecipitated using anti-ISG15 serum, thoroughly washed and immunocomplexes analysed by SDS-PAGE and subjected to immunoblotting with antiserum to E3 or ISG15. B. PKR+/+ cells were treated as above and cell extracts were collected at 16 hpi and immunoprecipitated using anti-E3 (with or without RNase treatment (10 µg for 15 min at room temperature); or without antibody; or using a pre-immune serum, thoroughly washed and immunocomplexes analysed by SDS-PAGE and subjected to immunoblotting with antiserum to E3 or ISG15. C. PKR+/+ MEFs treated as in A were fixed at 16 h.p.i. and processed for immunofluorescence analysis by confocal microscopy using antibodies directed against ISG15 (red), E3 (green), and TOPRO for staining nuclei (blue). Merged images are presented in the lower panels. Cells were visualized by confocal immunofluorescence microscopy. D. Immunoprecipitation and immunoflurescence of PKR−/− MEFs was performed as in A or in C.

It has been described that the influenza B virus protein NS1B inhibits ISGylation after binding through its amino terminal domain to ISG15 [27],[43]. To test whether E3 protein, that contains a similar domain to NS1, is able to bind to ISG15 protein, we performed immunoprecipitation (IP) assays in PKR+/+ cells using the following different viruses: WR, MVA, VVΔE3L lacking the entire E3L gene, and two deletion mutants VVE3LΔ83N and VVE3LΔ26C with truncated versions of E3L gene at the N and C-terminus [44]. After ISG15 IP, the entire E3 protein binds efficiently to ISG15 and the N-terminal mutant Δ83N, that lacks 83 aminoacids and the PKR binding domain, binds efficiently to ISG15 (Fig. 5A). In contrast, the C-terminal mutant Δ26C, that lacks 26 aminoacids and the ability to bind dsRNA, does not bind ISG15 (Fig. 5B). We also performed the reverse IP using an anti-E3 antibody and only the E3 protein that lacks the C- terminus fails to be immunoprecipitated (Fig. 5B, left panel). When IP was performed without antibody or using a pre-immune serum as a control, no interaction was observed (Fig. 5B, central panels). To analyze if RNA was involved in E3/ISG15 interaction, we treated the IP complex with RNase just before its loading in the SDS-PAGE, and found that the complex was destroyed, as no interaction was observed with any of the E3 proteins from the different viruses (Fig. 5B, left panel). This result indicate that RNA, and probably dsRNA, has a role as a linking component in the interaction between E3 and ISG15 proteins as its degradation abolishes the binding of both proteins. ISG15/E3 protein interaction was confirmed by confocal microscopy, as WR-, or MVA- or VVE3LΔ83N-infected MEFs showed co-localization between ISG15 and E3, while VVΔE3L- or VVE3LΔ26C-infected MEFs did not (Fig. 5C). In addition, we also studied if the presence of PKR was relevant for this interaction by performing both IP and confocal experiments in PKR−/− cells. Both approaches indicate that the interaction between ISG15 and E3 is independent of PKR, as in its absence the entire E3 and the protein that lacks the amino terminus are able to interact with ISG15 (Fig. 5D). The findings of Fig. 5 reveal that ISG15 binds the E3 protein in a PKR-independent manner and that binding requires the C-terminal domain of E3 spanning the RNA-binding site, which suggests that dsRNA acts as a linker.

### Enhanced susceptibility of ISG15 KO mice to VVΔE3L infection

**Figure 6:**
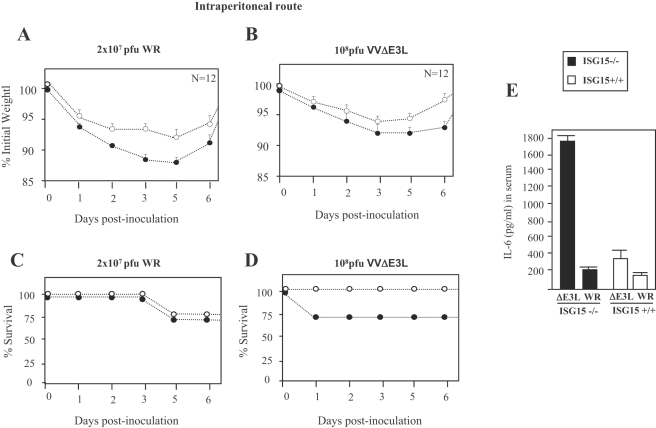
Virulence of WR or VVΔE3L after infection of ISG15+/+ and ISG15−/− mice. A–B. Mice were inoculated i.p with purified VACV (2×10^7^ PFU/mouse) or VVΔE3L (10^8^ PFU/mouse). Mice were individually weighed daily, and mean percentage weight loss of each group (n = 12) was compared with the weight immediately prior to infection. C–D. Survival rate after i.p inoculation with both viruses. Dead animals were scored daily and represented as the percentage of surviving animals. P≤0.01 in all experiments. E. IL-6 measured by ELISA from serum collected at 3 hpi. Results represent the mean±SD of pooled samples from 6 mice.

To further show that ISG15 is a biological relevant antiviral molecule against VACV infection, we next evaluated the susceptibility of ISG15−/− mice to the virus. Thus, we infected by the intraperitoneal (i.p) route ISG15−/− or ISG15+/+ mice with WR at 2×10^7^ or with the attenuated VVΔE3L at 10^8^ PFU/mouse and scored for prominent indicators of viral pathogenesis (weight loss and mortality). While there was reduced weight loss in ISG15−/− mice, survival was similar between both groups (Fig. 6, panel A and B). However, after VVΔE3L infection the ISG15−/− mice displayed signs of disease within 2 days, characterized by ruffled fur and lack of activity, and 25% of the animals died within 1 to 2 days. Half of the mice infected with VVΔE3L appeared sick at 4 days p.i., and 75% recovered after 7 to 8 days p.i. (Fig. 6C–D). We did not observe virus yields for ISG15−/− or ISG15+/+ mice infected with VVΔE3L in liver or spleen, while virus titers were easily obtained in WR infected mice (not shown).

Since the inflammatory response might explain the rapid signs of illness in VVΔE3L infected ISG15 KO mice, we measured serum cytokine levels (IL-6, TNF-α, IL-10, MCP-1, IFN-γ, and IL-12 p 70) at early times post infection. In ISG15+/+ mice, IL-6 levels were similar in serum from WR- or VVΔE3L- or mock-infected mice (Fig. 6E). In contrast, ISG15−/− mice infected with VVΔE3L had an 8-fold increase in serum levels of IL-6 compared with those infected with WR (P<0.01; Fig. 6E). There were no changes in levels of other cytokines analyzed between the groups (not shown).

**Figure 7:**
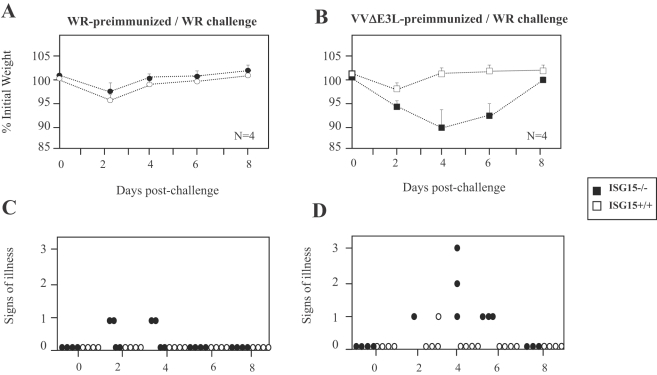
Evaluation of extent of protection of WR or VVΔE3L pre-immunized ISG15+/+ and ISG15−/− mice after challenge with WR. Four mice per group were first i.p, immunized with purified WR (2×10^7^ PFU/mouse) or VVΔE3L (10^8^ PFU/mouse), and 30 days later animals were i.n. challenged with 2×10^7^ PFU/mouse of purified WR. A–B. Mice were individually weighed daily, and mean percentage weight loss of each group (n = 4) was compared with the weight of mice taken prior to the booster. C–D. For each mouse signs of illness, such as ruffled fur and lack of activity, were monitored with the time and are given in arbitrary units.

We also examined the extent of protection conferred in animals pre-immunized with WR or VVΔE3L by i.p route. Thus, pre-immunized mice (as in Fig. 6) were challenged by i.n route with WR at 2×10^7^ PFU/mouse. In the case of ISG15+/+ and ISG15−/− mice pre-immunized mice with WR, the challenge had little effect on weight loss and signs of illness, clear signs of protection (Fig. 7A–B). However after WR challenge, VVΔE3L pre-immunized ISG15−/− mice developed weight loss and signs of sickness which was not observed in ISG15+/+ mice (Fig. 7C–D). These findings revealed a reduced protection to challenge with WR conferred by VVΔE3L pre-immunized KO mice, indicating limited adaptive immune response triggered by VVΔE3L infection of ISG15 −/− mice.

### The antiviral effect of ISG15 correlates with control of the inflammatory response after VACV infection

**Figure 8:**
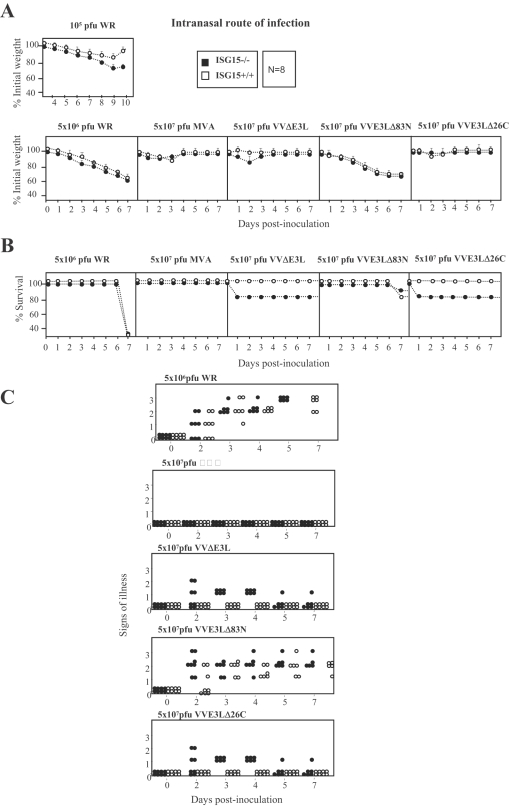
Virulence of different VACV strains after infection of ISG15+/+ and ISG15−/− mice. A. Mice were inoculated i.n with purified WR (10^5^ or 5×10^6^ PFU/mouse) or VVΔE3L (5×10^7^ PFU/mouse) or VVE3LΔ83N (5×10^7^ PFU/mouse) or VVE3LΔ26C (5×10^7^ PFU/mouse). Mice were individually weighed daily, and mean percentage weight loss of each group (n = 8) was compared with weight immediately prior to infection. B. Survival rate after i.n inoculation as described in A. Dead animals were scored daily and represented as the percentage of surviving animals. P≤0.01 in all experiments. C. Mice were inoculated as described above and for each individual signs of illness such as ruffled fur and lack of activity were monitored with time and are given in arbitrary units. P≤0.01 in all experiments.

Since VVΔE3L pre-immunized ISG15 KO mice developed disease transiently after i.n WR challenge and the upper respiratory tract is a natural route for variola virus infection, we next evaluated disease progression in ISG15+/+ and ISG15−/− mice after i.n inoculation with WR and several E3L deletion mutants (VVΔE3L; VVE3LΔ83N and VVE3LΔ26C). The highly attenuated MVA strain was included as control. Infected mice were scored for prominent indicators of viral pathogenesis (weight loss, signs of illness and mortality). After WR or VVE3LΔ83N infection, no significant differences in weigh loss and signs of illness were observed between ISG15−/− and ISG15+/+ mice, although slight differences in weight loss were observed between both groups of mice when inoculated with a lower dose of WR (Fig. 8A, upper panel). Mortality was higher in mice infected with WR, as all mice died within 7 days in the case of WR, while infection with VVE3LΔ83N caused 25% mortality (Fig.8A–B). However, clear differences were observed after i.n. inoculation of VVΔE3L or VVE3LΔ26C. While ISG15+/+ mice did not show signs of illness at any times p.i, ISG15−/− mice infected with VVΔE3L or VVE3LΔ26C showed disease as revealed by clear signs of illness as soon as 2 days and 25% of the animals died. About half of the mice infected with VVΔE3L or VVE3LΔ26C appeared sick at 4 days p.i, but 75% of them recovered after 7 to 8 days p.i, (Fig. 8C).

**Figure 9:**
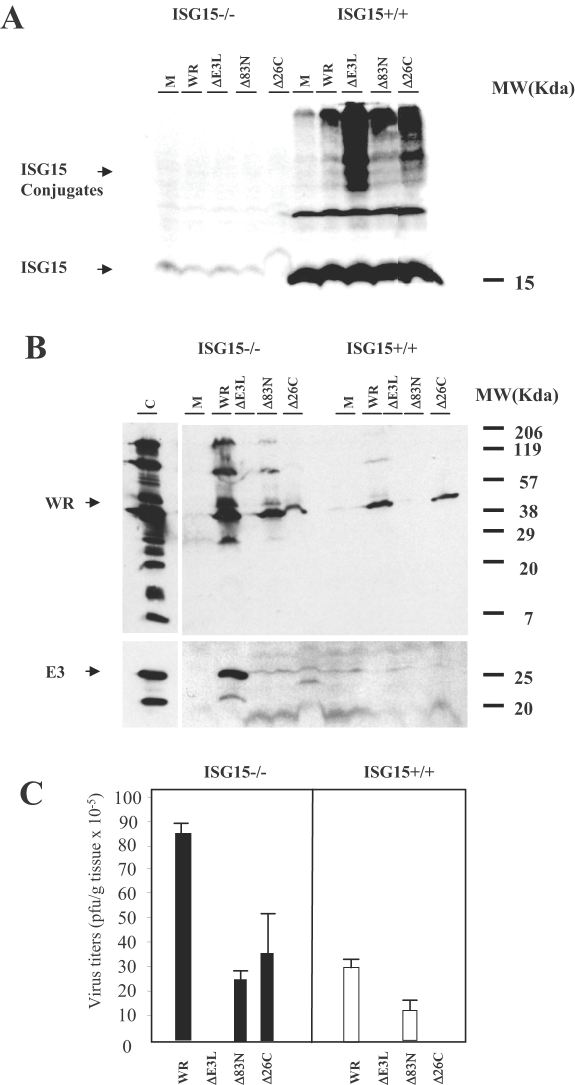
Viral replication of different VACV strains in infected ISG15+/+ and ISG15−/− mice. A–B. Western blot of ISG15 and of viral proteins in lung homogenates from ISG15+/+ and ISG15−/− mice. Animals were i.n, inoculated with purified WR (5×10^6^ PFU/mouse) or VVΔE3L (5×10^7^ PFU/mouse) or VVE3LΔ83N (5×10^7^ PFU/mouse) or VVE3LΔ26C (5×10^7^ PFU/mouse) and immunoblots reacted with specific antibodies to ISG15 and to viral proteins. Each sample represents pools from 2 mice per group. C. Lung homogenates were titrated by plaque assay in BSC40 (for WR) or by immunostaining in BHK-21 cells (for VVΔE3L). Each sample represents pools from 2 mice per group.

To analyze the status of ISGylation in the infected mice, lungs were homogenized and conjugation of ISG15 to its target proteins was determined by Western blot. While, ISG15−/− mice do not express ISG15 (Fig. 7A) and lungs homogenates from ISG15+/+ mice had similar amounts of the ISG15 protein, conjugation of ISG15 to its targets proteins is enhanced in lung extracts from mice infected with VVΔE3L or VVE3LΔ26C (Fig. 9A). Similar result was also observed in MEFs infected *in vitro* with VVΔE3L where high levels of ISG15 conjugates were clearly observed (Fig. 1A). These findings suggest that E3 blocks conjugation of ISG15 to its target proteins by its carboxy-terminal domain.

The presence of VACV proteins, as determined by Western blot, was more evident in lungs of ISG15−/− in comparison to ISG15+/+ mice infected with WR or the deletion mutant viruses (Fig. 9B). As expected, appearance of virus in lungs correlated with the presence of viral proteins in these tissue extracts (Fig. 9B–C). These results indicate that although the absence of ISG15 has no effect in the mortality of the mice at a high dose of WR inoculation (5×10^6^ PFU/mice; Fig. 8A, middle and lower panel), it has an effect in the replication of the WR and E3L mutant viruses, as seen by the different amount of viral protein and virus titers in lung tissues of ISG15−/− versus ISG15+/+ mice (Fig. 9B–C).

**Figure 10:**
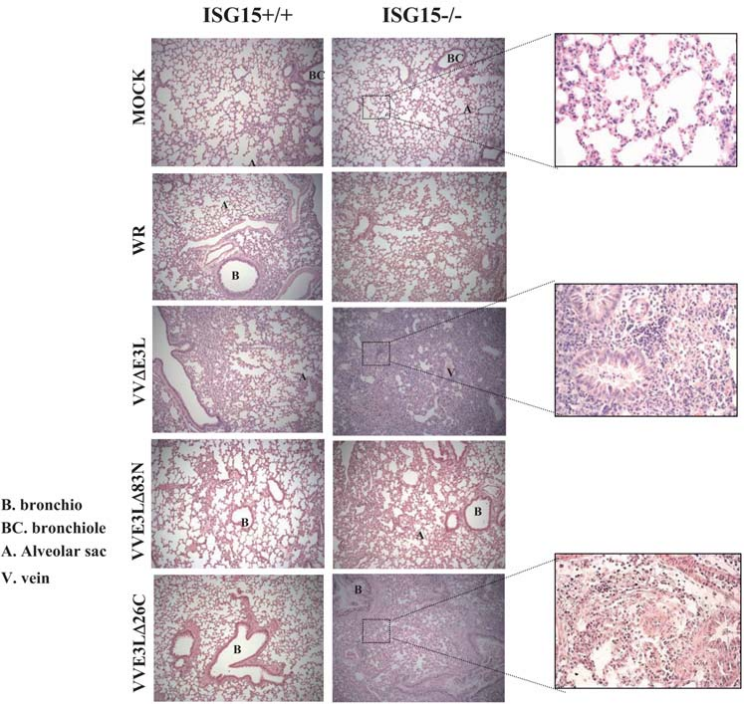
Histopathology of lungs from ISG15+/+ and ISG15−/− mice intranasally infected with VACV mutant viruses. Lungs from ISG15+/+ and ISG15−/− mice i.n inoculated with, WR (5×10^6^ PFU/mouse) or MVA (5×10^7^ PFU/mouse) or VVΔE3L (5×10^7^ PFU/mouse) or VVE3LΔ83N (5×10^7^ PFU/mouse) or VVE3LΔ26C (5×10^7^ PFU/mouse) were resected, sectioned and stained with hematoxilin and eosin. For each group of animals representative fields are shown at a magnification of 40× (left panels) and 100× (right panels). B. Bronchio, BC. Bronchiole, A. Alveolar sac, V. Vein.

Histological examination of lung tissue showed that ISG15+/+ animals infected with the different mutant viruses had no inflammatory cells infiltrating the lung parenchyma. In contrast, lung sections obtained from ISG15−/− mice infected with VVΔE3L or VVE3LΔ26C presented severe inflammation with alveolar wall thickening and infiltration of inflammatory cells (see enlarged sections in Fig. 10). This phenotype was not observed in WR- or VVE3LΔ83N- infected ISG15−/− mice (Fig. 10). This result indicates that in ISG15−/− mice, pathogenesis and development of an inflammatory response is mediated by the absence of E3 virus expression. This phenotype was maintained after VVΔE3L and VVE3LΔ26C pointing to E3 as a major VACV molecule involved in virus evasion of the IFN-defense ISG15 antiviral protein.

## Discussion

Pro-inflammatory and IFN-stimulated genes (ISGs) represent essential components of the innate immune response to viral infection (40). Upon viral entry into cells, ISG induction occurs in two waves: acute, IFN-independent induction of a subset of ISGs and delayed, IFN-dependent induction via the production of IFN-α/β during the initial phase. In many viral infections, IFN-independent ISG induction is mediated by the IRF-3 phosphorylation, homodimerization, and nuclear translocation. Activated IRF3, in turn, induces the expression of type I IFN genes, whose products trigger strong induction of a subsets of ISGs, including IFN-β which after its release and ligand-binding to its receptor then initiates IFN-dependent ISG induction via the IFN receptor and JAK/STAT signaling pathways. IFN-inducible enzymes, like the 2.5 OAS/RNAse L system, PKR, and M×, are the best characterized proteins that mediate antiviral action of IFN. Another protein, ISG15, was first identified as an IFN-stimulated gene whose expression is induced strongly by IFN-α/β treatment and can be detected at low constitutive levels in cells [45]. ISG15 modifies several important molecules and affects type I IFN signal transduction; ISG15 expression is markedly increased following viral infection (14, 30, 49), and many viruses encode inhibitors of the IFN-transduction pathway or specific inhibitors of ISG to avoid deleterious effects triggered by these cytokines.

Among animal viruses, the poxvirus family contains a large array of genes which are used by the virus to evade host immune responses. VACV encodes multiple proteins that interfere with complement regulatory proteins, with many cytokines and chemokines, with TLRs (Toll like receptors) and signal transduction pathways, with apoptosis, and others [46]. One of the VACV proteins with strong inhibitory activity of IFN-induced pathways is E3 [47]. E3 represses the host cell antiviral response inhibiting both PKR and RNaseL, which trigger global inhibition of protein synthesis and virus replication [35],[36],[37]. In addition, E3 blocks the activation of IRF3 [40],[41], and effectively prevents the first wave of type I IFN synthesis. E3 has two domains, an N-terminal involved in the direct inhibition of PKR, its nuclear localization, and Z-DNA binding [34],[48],[49],[50], and the C-terminal that contains the dsRNA-binding domain required for IFN-resistance and for the broad host range phenotype of the virus [44],[51]. It has been described that VACV lacking E3 (VVΔE3L) replicates in PKR or RNaseL deficient cells [40].

Through the use of microarrays we identified the gene ISG15 as being induced in the course of infection of human cells with different strains of VACV [28],[29],[30]. The attenuated mutant VVΔE3L also produces an increase in ISG15 messenger levels [31]. The reason for the induction of ISG15 mRNA levels by attenuated viruses (Fig. 1) is probably due to the activation of several cellular signal transduction cascades and of host transcription factors [28],[30],[31]. Since MVA and NYVAC strains contain the E3L gene, this upregulation may be independent of E3 expression with induction being likely due to the increase in IFN-β levels.

In this study we showed that a VACV mutant lacking E3, which cannot grow in ISG15 WT cells, is able to replicate both in MEFs cells derived from ISG15 KO mice or in ISG15 silenced cells. In addition viral titers also increase in the absence of ISG15 indicating that ISG15 has an essential role against infection of VACV. During infection of MEFs from ISG15 KO or ISG15 depleted cells, the presence of E3 enhances viral production since the WR titers are greater than those after infection with the VVΔE3L mutant virus. One explanation of this phenotype is that the mutant virus lacking E3 triggers apoptosis through PKR activation which, in turn, reduces virus production as previously described [52]. The role of ISG15 in VACV replication was also supported by the more abundant VACV infectious virus and viral proteins produced in lungs of ISG15−/− mice compared with lungs of ISG15+/+ mice after infection with WR or with the E3L deletion mutant viruses (Fig. 9B–C).

While the depletion of ISG15 has an effect on VVΔE3L mutant phenotype and restores virus growth, over-expression of ISG15 in ISG15−/− murine cells using a retroviral transduction system revert the restricted VVΔE3L viral growth. Over-expression of ISG15 also reduces markedly WR titers reinforcing the idea that ISG15 plays a role in the control of VACV replication.

Inhibition of ISG15 function by VACV is likely due to its interaction with VACV E3, as shown by IP and confocal analyses. This interaction with ISG15 occurs independently of PKR, through the C-terminal region of E3 and requires RNA. We have shown that ISG15 controls the *in vitro* replication of VACV in a PKR-independent manner, as WR and VVΔE3L titers do not increase in murine PKR−/− cells in comparison to those observed in PKR+/+. While in murine cells VVΔE3L is able to replicate in a PKR-independent manner, as also described in MEFs lacking RNase L [53], in human HeLa cells with PKR expression suppressed by siRNA, the mutant virus is able to grow [40]. The differences in cell origin might explain the distinct effect of the IFN system in the control of VACV replication.

The mutant VVΔE3L virus that was able to replicate in ISG15−/− MEFs (Fig. 2) did not replicate in ISG15 KO mice (Fig. 7), but surprisingly infection with VVΔE3L provokes sickness and mortality only in ISG15−/− mice. This was probably related to the strong inflammatory response triggered by the mutant in ISG15 KO mice, as observed by the increased levels of IL-6 in serum (Fig. 4E). Although the biological relevance of this observation remains to be established, it can be suggested, in view of the functions assigned to ISG15 in the innate immune response [54], that this molecule plays a role as regulator of IFN-triggered innate responses during VACV infection. It will be of interest to know the type of innate response triggered in ISG15 KO mice infected with VVΔE3L.

The inability of VVΔE3L to cause significant disease in WT mice is presumably due, at least in part, to induction of type I IFN that, in turn, leads to up-regulation of antiviral proteins, such as PKR and 2–5 OAS/RNaseL system. The mutant virus that lacks the C terminus of E3L gene involved in the dsRNA sequestration (VVE3LΔ26C) is completely attenuated in WT mice; however deletion of the N terminus (VVE3LΔ83N) reduces pathogenesis 500- to 5,000-fold [33],[48]. We extended these *in vivo* studies using the i.n route and compared WR and E3L mutant viruses in WT and ISG15 KO mice. We observed that only after VVΔE3L or VVE3LΔ26C inoculation (i.n), the mortality of mice was increased by the absence of ISG15 (Fig. 6). In the case of WR or VVE3LΔ83N, there were no differences in mortality of both viruses in ISG15 KO in comparison to WT mice although viral replication was enhanced in the lungs of ISG15−/− in comparison to ISG15+/+ mice. One explanation is that ISG15 is made non functional after infection with these viruses, because the carboxy-terminal domain of E3 binds to ISG15 and blocks its activity. These observations are in correlation with the reduced presence of conjugates in lungs of WT mice infected with WR or VVE3LΔ83N, compared with infection by VVΔE3L or VVE3LΔ26C. Although lung homogenates presented similar amounts of ISG15 protein, the conjugation of ISG15 to its targets proteins, was greatly enhanced after infection with VVΔE3L or VVE3LΔ26C (Fig. 7). This evidence suggests that inhibition of conjugation of ISG15 is mediated by E3 and this inhibition requires the presence of the dsRNA binding domain. Similar result was also observed in MEFs infected *in vitro* with VVΔE3L where high levels of ISG15 conjugates were clearly observed (Fig. 1A). The cause of mortality of ISG15 KO mice after infection with VVΔE3L or VVE3LΔ26C was a massive inflammation of lungs with alveolar wall thickening and infiltration of cells (Fig. 8). These results indicate a role of ISG15 in the control of an inflammatory response by regulating cytokine levels.

Cytokine and chemokine release occurs rapidly in response to virus infection, with the aim of recruiting inflammatory leukocytes in order to limit virus replication and spread, and to induce adaptive immunity. However, prolonged expression of chemokines in the context of viral infections may be detrimental to the host. We find that in the absence of ISG15, infection with VVΔE3L produces an increase of IL-6 that correlates with short-term morbidity and complications that include pulmonary function abnormalities. Although the mechanisms of this up-regulation remains to be established, it can be speculated in view of the functions assigned to ISG15 that it might be involved in the regulation of cytokine signal transduction, through the stabilization of specific signalling components that facilitate the development of a correct innate immune response. In this sense a family of intracellular proteins called suppressors of cytokine signalling (SOCS) are essential for the regulation of cytokine expression having a critical role in the regulation of the innate response. Considering that SOCS-1 and SOCS-3 negatively regulate the IFN-induced signal cascade, and VACV E3 protein inhibits the type I IFN response, it is possible that E3 or other viral proteins may regulate the IFN response by affecting SOCS protein expression regulating the ISG15 activity by an unknown mechanism. We have previously demonstrated that although WR provokes a general downregulation of cellular mRNAs, there are a discrete number of human genes that are induced selectively during the course of VACV infection. A variety of these upregulated genes encode different members of the SOCS family [29] indicating that probably VACV may modify SOCS protein expression to manipulate the cytokine pathway and the antiviral host response. This strategy may be used to reduce the efficacy of innate and acquired immune responses to infection. However, WR modification of cytokine or chemokine responses may also be a mechanism to recruit new targets for infection, or provide new niches for infection. It has been described that over-expression of HCV core protein inhibits IFN signalling and induces SOCS-3 expression. SOCS-1 and SOCS-3 proteins have been reported to inhibit IFN-induced activation of the JAK-STAT pathway and expression of antiviral proteins, such as M×A [55].

There are similarities between the functions of VACV E3 and the NS1 dsRNA-binding protein of influenza virus. NS1 blocks IRF3 phosphorylation and IFNβ mRNA induction [56]. In addition, NS1 is an inhibitor of PKR, suggesting that dsRNA sequestration is a strategy used by both RNA and DNA viruses to evade the IFN induction and action [57]. Furthermore NS1B binds and blocks ISG15 protein inhibiting the ISGylation. The region of the NS1B protein that is required for this inhibition includes the domain that binds dsRNA. VACV may have a similar mechanism of influenza NS1 to evade ISG15 action as well.

We conclude that the cellular ISG15 protein has an essential role in VACV replication, acting as a negative feedback regulator of the cytokine signalling pathway and regulating in this way the innate response. VACV has therefore developed a mechanism to counteract this antiviral host response through E3. Because VVΔE3L is not lethal to ISG15+/+ mice lacking PKR, RNase L, and M×1 [40], there must be an additional IFN-induced antiviral pathway(s) effective against viruses, in which ISG15 should play an essential role. Understanding the host responses triggered by ISG15 and virus mechanisms of escape is necessary for development of therapies against important human pathogens.

## Materials and Methods

### Cells, viruses and infection conditions

HeLa cells (ATCC) were cultured in Dulbecco's medium (DMEM) supplemented with 10% newborn bovine serum (NCS) and antibiotics (Gibco, http://www.invitrogen.com). ISG15−/− cells and their wild type counterpart were generated by Osiak et al [58] and cultured in DMEM with 10% fetal calf serum (FCS). PKR−/− and their wild type counterpart [59] were cultured in Dulbecco's modified Eagle's medium with 10% FCS. VACV wild-type Western Reserve strain (WR) was grown on monkey BSC-40 cells (African green monkey kidney cells), purified by sucrose gradient banding as described (24) and titrated in BSC-40 cells. MVA and NYVAC, as well as VACV mutant of E3L were grown in Baby hamster kidney cells (BHK-21), sucrose purified and titrated in BHK-21 cells by immunostaining as previously described [60]. VACV constructs deleted of E3L (VVΔE3L), of the first 83 N-terminal amino acids of E3L (VVE3LΔ83N), or of the last 26 C-terminal amino acids of E3L (VVE3LΔ26C) were kindly provided by B. L. Jacobs (University of Arizona, USA) [44],[61].

### Quantitative Real-Time RT-PCR

RNA (1 µg) was reverse-transcribed using the Superscript first-strand synthesis system for reverse transcription-PCR (RT-PCR) (http://www.invitrogen.com). A 1∶40 dilution of the RT reaction mixture was used for quantitative PCR. Primers and probe sets used to amplify ISG15 was purchased from (http://www.appliedbiosystems.com). RT-PCR reactions were performed according to Assay-on-Demand, optimized to work with TaqMan Universal PCR MasterMix, No AmpErase UNG, as described [28]. All samples were assayed in duplicate. Threshold cycle (Ct) values were used to plot a standard curve in which Ct decreased in linear proportion to the log of the template copy number. The correlation values of standard curves were always >99%.

### Cellular viability assay

Cells were grown in 96-well plates to confluency and infected with different VACV or VVΔE3L viruses at the indicated multiplicity of infection (MOI) from 0.01 to 10 PFU/cell. At 24 hours post-infection (hpi), the medium was removed and cytolysis was determined by crystal violet staining as described previously [62]. The percentage of viable cells was calculated assuming the survival rate of uninfected cells to be 100%.

### Immunoblotting

Murine embryonic fibroblasts (MEFs) were infected in 6-well plates with WR, or MVA, or NYVAC, or VVΔE3L and collected at indicated hpi in lysis buffer (50 mM Tris-HCl pH 8.0, 0.5 M NaCl, 10% NP40, 1% SDS). Equal amounts of protein lysates (100 µg) were separated by 14% or 8% SDS-polyacrylamide gel electrophoresis (SDS-PAGE), transferred to nitrocellulose membranes and incubated with antibodies, anti-ISG15 [58], -actin (http://www.sigmaaldrich.com), -E3 (kindly provided by B.L. Jacobs) followed by peroxidase-conjugated mouse or rabbit secondary antibodies. For the *in vivo* measurement of ISG15, parental or ISG15−/− mice were infected with WR, VVΔE3L, VVE3LΔ83N or VVE3LΔ26C at the multiplicity indicated. Lung samples were homogenized and mixed with SDS loading buffer and boiled for 10 min before Western blot analysis. ISG15 expression was detected as previously described with a rabbit antiserum against ISG15 [58] followed by peroxidase-conjugated rabbit secondary antibodies. Blots were developed using ECL (http://www.amersham.com).

### Immunoprecipitation analysis

Confluent MEF cells grown in 100 mm plates were treated with mouse IFN-α (100 units/ml) during 10 hrs and infected at 3 PFU/cell for 16 h with the recombinant viruses indicated and cells were collected and lysed and clarified supernatant was incubated with 20 µg of anti-mouse IP beads (http://www.ebioscience.com) previously incubated with a rabbit antiserum against ISG15 [58] or against E3 respectively. Immunoprecipitates were analyzed by SDS-PAGE followed by immunoblot with the antibody anti-E3 (kindly provided by B.L. Jacobs). The RNase treatment consisted in an incubation of the IP extracts with 10 µg of RNAse for 15 min at room temperature.

### Viral inoculation of mice and sample collection

The origin of ISG15−/− mice has been described [58]. ISG15−/− and control wild type (WT) C57/BL-6 mice ( 6 to 10 weeks old) were immunized i.n in 25 µl PBS with VACV at 5×10^6^ or 10^5^ PFU/mouse or with VVΔE3L or VVE3LΔ83N or VVE3LΔ26 at 5×10^8^ PFU/mouse. The i.p inoculation was with VACV at 2×10^7^ or VVΔE3L at 10^8^ PFU/mouse in 200 µl PBS. Animals were sacrificed at various times post-inoculation and spleen, liver, ovaries and lungs were removed, washed with sterile PBS, and stored at −70°C. Serum was obtained by retro-orbital bleedings 3 hours post-inoculation and was allowed to clot 1 hour at 37°C; after leaving samples at 4°C overnight, they were spun down in a microcentrifuge, and serum removed and stored at −20°C.

### Cytokine Analysis

Secreted IL-6 from serum of mice infected i.p with WR or VVΔE3L at the indicated days was measured with the quantitative human IL-6 (BD Biosciences) according to the manufacturer's instructions. Captured IL-6 was quantified at 450 nm with a spectrophotometer. Triplicate samples were measured in two independent experiments. Alternatively, serum cytokine levels were analyzed for IL-6, TNF, IL-10, MCP-1, IFN-γ, and IL-12 p 70 by using the cytometric bead array mouse inflammation kit as indicated by the manufacturer (http://www.BDBiosciences.com).

### Transfection of ISG15 siRNA

ISG15-synthetic siRNA (s79221 and s79223), scrambled siRNA (used as a negative control; s4390843) and GAPDH siRNA (used as a positive control; s4390849) were purchased from Applied Biosystems and resuspended in RNase-free H_2_O. Transfection of siRNAs targeting each mRNA was carried out according to the manufacturer's instructions with some modifications. Murine ISG15+/+, PKR+/+ and PKR−/− embryonic fibroblasts were plated in 12-well plates 18 to 24 h before transfection. On the day of transfection, RNA-lipid complexes were introduced into each well of cells (20 nM RNA) by using siPORT Amine transfection reagent (http://www.ambion.com). The effect of specific siRNAs on target protein abundance was assessed by Western-blot. Twenty-four hours after transfection, siRNA-treated and non-treated control cells were mock-infected or infected with different WR or VVΔE3L viruses at 0.1 PFU/cell and CPE were visualized by phase-contrast microscopy at the indicated times p.i.

### Ectopic ISG15 protein expression by retroviral transduction

ISG15+/+ and ISG15−/− MEFs were transduced with high-titer viral supernatants corresponding to the pISG15-ires-GFP retroviral vector obtained as described [63]. Supernatants were collected at 48 h after transfection, filtered through a 0.45-µm-pore-size filter, and supplemented with complete DMEM medium +10% FCS before addition to growing MEFs. This protocol was repeated each 12 hours three times in presence of polybrene. The transduction efficiency was evaluated by Western-blot. Twenty-four hours after retroviral infection treated and non-treated control cells were mock-infected or infected with different VACV or VVΔE3L viruses at 0.1 PFU/cell and and CPE were visualized by phase-contrast microscopy at the indicated times p.i.

### Immunofluorescence

PKR+/+ and PKR −/− embryonic murine fibroblasts cultured on coverslips were infected with the viruses indicated. At 16 hpi cells were washed with phosphate-buffered saline (PBS), fixed with 4% paraformaldehyde (PFA) and permeabilized (10 min, room temperature) with 0.1% Triton X-100 in PBS, washed, and blocked with 20% bovine serum albumin (BSA) in PBS. Cells were incubated (1 h, 37°C) with anti-ISG15, -E3 (mouse antibody kindly provided by B. Moss); coverslips were washed extensively with PBS and further incubated (1 h, 37°C) with ToPro (http://www.molecularprobes.com) and appropriate fluorescein- or Texas Red-conjugated isotype-specific secondary antibodies. After washing with PBS, coverslips were mounted on microscope slides using Mowiol (http://www.calbiochem.com). Images were obtained using a Bio-Rad Radiance 2100 Confocal Laser microscope (http://www.biorad.com).

### Immunohistochemistry

Formalin-fixed lung from mice mock-infected or infected with WR, VVΔE3L, VVE3L•83N or VVE3L•26 was resected, sectioned and stained with both hematoxilin and eosin as previously described [64].

## Supporting Information

Figure S1 Effect of ISG15 overexpression on virus citotoxicity after infection of MEFs with virulent and E3L deletion VACV mutant viruses. A–B. ISG15−/− or ISG15+/+ MEFs were transduced with high-titer viral supernatants corresponding to the pISG15-ires-GFP retroviral vector. CPE was visualized by phase-contrast microscopy at the indicated times p.i.
(0.68 MB PDF)

Table S1 Levels of ISG15 mRNA detected by quantitative real-time RT-PCR after infection of HeLa cells with several VACV mutants.
(0.02 MB PDF)
