# Supplementary figures and images for: Vaccinia Virus E3 Protein Prevents the Antiviral Action of ISG15
Source: PLoS Pathog. 2008 Jul 4;4(7):e1000096. doi: 10.1371/journal.ppat.1000096 (PMC2434199; doi:10.1371/journal.ppat.1000096)

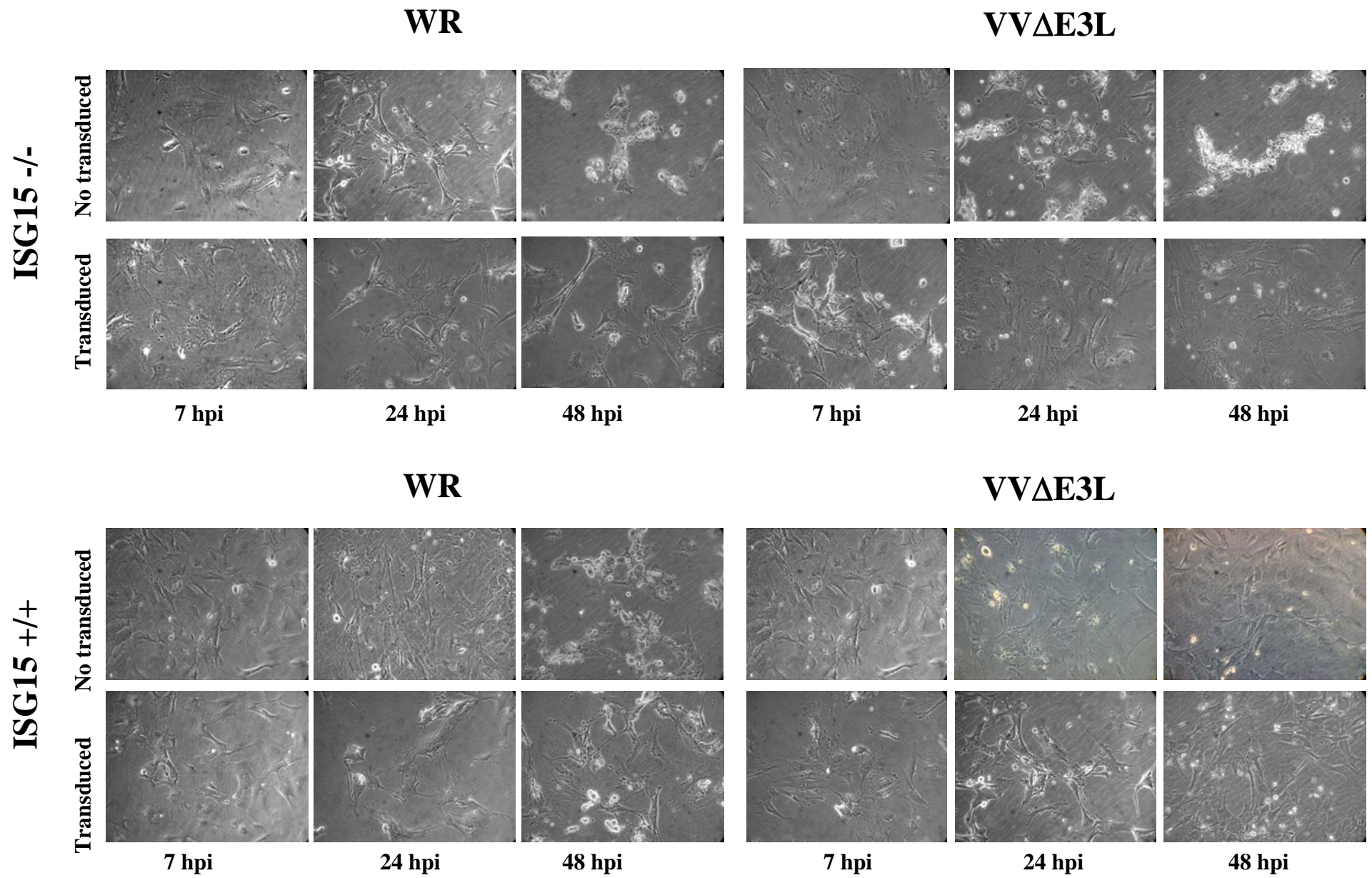

SuppFigure 1

Supplement: Figure S1 — Effect of ISG15 overexpression on virus citotoxicity after infection of MEFs with virulent and E3L deletion VACV mutant viruses. A–B. ISG15−/− or ISG15+/+ MEFs were transduced with high-titer viral supernatants corresponding to the pISG15-ires-GFP retroviral vector. CPE was visualized by phase-contrast microscopy at the indicated times p.i. (0.68 MB PDF) [file ppat.1000096.s001.pdf]
