# Supplementary material for: Vaccinia Virus E3 Protein Prevents the Antiviral Action of ISG15
Source: PLoS Pathog. 2008 Jul 4;4(7):e1000096. doi: 10.1371/journal.ppat.1000096 (PMC2434199; doi:10.1371/journal.ppat.1000096)
Supplement: Table S1 — Levels of ISG15 mRNA detected by quantitative real-time RT-PCR after infection of HeLa cells with several VACV mutants. (0.02 MB PDF) [file ppat.1000096.s002.pdf]

Supplementary table 1 . ISG-15 expression in different VV strains

Fold change by RT-PCR

	**t=2**	**t=6**	**t=16**
**WR**	0.16	0.60	0.49
**MVA**	5.09	4.9	4.6
**NYVAC**	0.96	5.9	5.7
**VVΔ3EL**	2.04	2.29	3.81
